# Impacts of US Bilateral Aid Disruptions on HIV Resurgence in Zambia: A Mathematical Modeling Study

**DOI:** 10.1093/ofid/ofaf511

**Published:** 2025-09-11

**Authors:** Lloyd B Mulenga, Kebby Musokotwane, Suilanji Sivile, Khozya D Zyambo, Roma Chilengi, Kennedy Lishimpi, George Sinyangwe, Sombo Fwoloshi, Chimika Phiri, Henry Phiri, Davies Kampamba, David J Kaftan, Sulani Nyimbili, Daniel T Citron, Hae-Young Kim, Anna Bershteyn

**Affiliations:** Ministry of Health, Lusaka, Zambia; University Teaching Hospital, Adult Infectious Diseases Center, Lusaka, Zambia; Division of Infectious Diseases, Department of Internal Medicine, School of Medicine, University of Zambia, Lusaka, Zambia; Division of Infectious Diseases, Department of Medicine, Vanderbilt University Medical Center (VUMC), Nashville, Tennessee, USA; Vanderbilt Institute for Global Health (VIGH), Vanderbilt University Medical Center (VUMC), Nashville, Tennessee, USA; National AIDS Council, Lusaka, Zambia; Ministry of Health, Lusaka, Zambia; University Teaching Hospital, Adult Infectious Diseases Center, Lusaka, Zambia; Division of Infectious Diseases, Department of Internal Medicine, School of Medicine, University of Zambia, Lusaka, Zambia; Ministry of Health, Lusaka, Zambia; University Teaching Hospital, Adult Infectious Diseases Center, Lusaka, Zambia; Zambia National Public Health Institute, Lusaka, Zambia; Ministry of Health, Lusaka, Zambia; Ministry of Health, Lusaka, Zambia; University Teaching Hospital, Adult Infectious Diseases Center, Lusaka, Zambia; Division of Infectious Diseases, Department of Internal Medicine, School of Medicine, University of Zambia, Lusaka, Zambia; Ministry of Health, Lusaka, Zambia; University Teaching Hospital, Adult Infectious Diseases Center, Lusaka, Zambia; Ministry of Health, Lusaka, Zambia; Ministry of Health, Lusaka, Zambia; Department of Population Health, NYU Grossman School of Medicine, New York, NY, USA; Department of Population Health, NYU Grossman School of Medicine, New York, NY, USA; Department of Population Health, NYU Grossman School of Medicine, New York, NY, USA; Department of Population Health, NYU Grossman School of Medicine, New York, NY, USA; Department of Population Health, NYU Grossman School of Medicine, New York, NY, USA

**Keywords:** bilateral aid, HIV, mathematical modeling, PEPFAR, Zambia

## Abstract

**Background:**

Of countries with high HIV prevalence, Zambia had the largest proportion of funding to its HIV program from the United States President's Emergency Plan for AIDS Relief (PEPFAR)—84% at the start of 2025. Abrupt withdrawal of bilateral aid in January 2025 disrupted HIV services. This study aimed to estimate the health and epidemiological consequences of the disruptions, and to what extent impacts could be mitigated by restoring services.

**Methods:**

We leveraged a previously developed HIV agent-based network transmission model, Epidemiological MODeling software for HIV, calibrated to Zambian HIV data at the provincial level. Health authorities leading the Zambian HIV program identified data and assumptions regarding impacts of aid disruptions by province and associated uncertainty ranges. We simulated disruptions lasting 3 months, 1 year, 4 years, or unabated, versus a counterfactual of no disruptions, over 2025–2060. Outcomes included additional HIV infections, deaths, and prevalence.

**Results:**

Unabated disruptions added 3.3 million HIV acquisitions (8.8x more than no disruption) and 1.6 million HIV deaths (5.3x), with the largest number among women (1.5 million acquisitions, 790 933 deaths) and the largest proportional increase among children (21.6x acquisitions, 20.8x deaths). Restoring services within 3 months would limit additional acquisitions to 54 863 (+13.1%) and additional deaths to 32 550 (+8.7%). HIV prevalence would increase by 4.5x if disruptions were unabated through 2060, but would not change (0.0x) if services were restored within 3 months.

**Conclusions:**

Rapid restoration of HIV services disrupted by the 2025 bilateral aid withdrawal could save >1.5 million lives and prevent epidemic resurgence in Zambia.

## Teaser

Rapid restoration of HIV services disrupted by 2025 bilateral aid withdrawal in Zambia could save >1.5 million lives and prevent a quadrupling of HIV prevalence. The most lives at risk are among women, and largest fold change anticipated among children.

Among countries with high HIV prevalence, Zambia is particularly vulnerable to changes in foreign aid. In 2024, 84% of Zambia's HIV financing came from the United States President's Emergency Program for AIDS Relief (PEPFAR)—one of the largest proportions globally [1]. While the majority of Zambia's health sector financing is domestically sourced, accounting for 92% of the Ministry of Health's budget [2], HIV programs continue to receive substantial external funding. This reflects the long-standing and complementary role of international partners in supporting the national HIV response, particularly for treatment and prevention services. These funds are granted through annual cooperative agreements with the Zambia Ministry of Health and also through nongovernmental organizations such as implementing partners, civil society organizations, and community-based organizations [3]. The last HIV care continuum population-based survey in 2021 showed Zambia nearly reaching UNAIDS “95–95–95” treatment targets in 2021 by diagnosing 89% of people living with HIV (PLHIV), providing treatment to 98% of those diagnosed, and enabling 96% of those on treatment to achieve undetectable HIV viral load, while more recent model-based estimates showed Zambia exceeding the “95–95–95” targets in 2024 [4, 5]. At the start of 2025, Zambia was providing treatment to 1.3 million PLHIV, with 42% of its antiretroviral medications purchased by PEPFAR [6]. In addition, Zambia has achieved an estimated 892 572 initiations of oral pre-exposure prophylaxis (PrEP) as of the fourth quarter of 2024, and in February 2024, with PEPFAR support, Zambia had become the first country outside the United States to provide long-acting injectable cabotegravir for PrEP outside of a research study [7, 8]. Thanks in large part to these successes, life expectancy in Zambia rose from 39 years in 2000 to 61 years in 2024 [9].

Beyond its financial contribution, PEPFAR directly employed thousands of front-line health workers providing HIV treatment and prevention directly to people living with or at risk of HIV, as well as staff supporting healthcare operations such as appointment scheduling, electronic health record-keeping, outreach to clients who missed appointments, mentoring and supervision to clinical staff, logistics and supply chain management, distribution and warehousing of HIV medications and supplies, laboratory specimen transport, and data monitoring and forecasting [10]. PEPFAR-supported employees of the United States Agency for International Development (USAID) played a particularly large role in 5 of Zambia's 10 provinces (Luapula, Copperbelt, Northern, Muchinga, and Central Provinces), where USAID-employed healthcare workers provided direct clinical care in ∼70% of facilities—40% predominantly USAID-staffed and 30% co-staffed by USAID and Zambian government-employed providers [11]. Though all facilities had implemented some degree of hybrid implementation with the Zambian government, the important role of PEPFAR meant that full program hand-off to the government would require advance planning and systematic, staged withdrawal in order to avoid severe service disruptions.

On 20 January 2025, an executive order from the President of the United States re-evaluating and realigning foreign aid prompted an abrupt withdrawal of foreign aid workers and other resources in Zambia. A “stop-work” order resulted in the immediate termination of USAID employees, including direct HIV care providers [12]. Zambian government-employed health workers were directed to undertake additional HIV clinical duties to the extent possible, but this was insufficient to backstop a drastic decrease in the HIV care workforce. Since the initial executive order, a waiver has permitted continued funding and human resources for certain life-saving services, including HIV treatment, prevention of mother-to-child transmission of HIV services, HIV testing, and supply chain [13]; however, USAID-staffed clinics remained closed, most HIV prevention services ceased, and healthcare operations continued to suffer from disrupted logistics, supply chains, data systems, and other critical functions. Contracts for implementing partners (IPs) responsible for drug and commodity distribution were terminated, and procurement orders for testing reagents and point-of-care cartridges, which were nearing delivery, were halted. The sample courier system, four-fifths of which was funded by PEPFAR, collapsed due to the withdrawal of IPs who covered the costs of fuel, courier, and laboratory personnel at central labs, resulting in severe local stockouts and inability to process specimens [14]. US Centers for Disease Control and Prevention (CDC) funded clinics were also affected by the stop-work order, which led to the suspension of CDC-supported clinical staff and the interruption of key services such as HIV testing, laboratory diagnostics, and surveillance systems.

For example, electronic medical record systems, which had been run with PEPFAR support, became inaccessible, including in clinics that had implemented a fully digital workflow without paper-based records on-site. This occurred because data clerks were terminated and typically instructed to return laptops to the implementing partner's headquarters; others left computer hardware on-site but without the opportunity to transfer passwords, accounts, or informatics skills to other staff [15]. As a result, clinics were unable to provide appointments, contact to clients who missed appointments, distinguish new versus returning clients, or determine what medications had been previously prescribed to returning clients. This posed major challenges for retaining PLHIV in care. Suspension of support from IPs further undermined HIV services, especially HIV prevention. For example, 32 wellness centers providing HIV services to more than 20 000 vulnerable populations across 7 of Zambia's 10 provinces ceased operations. Similarly, DREAMS centers, which offered comprehensive support to adolescent girls and young women in 21 districts, and 16 standalone centers providing voluntary medical male circumcision, were shut down [16].

As leaders at the Zambia Ministry of Health and our research collaborators, we sought a quantitative understanding of the health and epidemiological impacts of bilateral aid disruptions and to what extent impacts could be mitigated through timely restoration of services. This study aimed to assess the impacts of aid disruption leveraging a previously developed mathematical model of HIV transmission, care, and prevention in Zambia.

## METHODS

### Model Description

Our study leveraged a previously developed HIV transmission model, EMOD-HIV [17]. The main components of the model are demographics, HIV transmission, disease progression, and HIV treatment and prevention services.

Demographically, the model is comprised of individual agents distributed geographically across 8 regions representing Zambia's 10 provinces. Two provinces—Muchinga and Northern, which collectively contain <8% of all PLHIV in Zambia—were combined with geographically contiguous Luapula Province in order to simplify the model for computational efficiency. After initializing the population, the model created new agents born to mothers and removed individuals through either HIV-related mortality or HIV cause-deleted “background” mortality rates [18]. Fertility and mortality rates were obtained from the age-/sex-specific mortality estimates by the UN Population Division (Supplementary Tables 1–4) [19].

HIV in the model could be acquired vertically (from mother to child) or sexually through a relationship network [20, 21]. Populations are stratified into 3 sexual risk groups (eg, low, medium, and high), with age- and risk-assortative mixing calibrated to sexual behavior data using population-based HIV surveillance studies. In relationships, HIV transmission probability at each coital act was determined by whether a condom was used, whether either individual had a sexually transmitted infection, whether the HIV-positive partner had suppressed HIV viral load through HIV treatment, and whether the HIV-negative partner used PrEP or had received voluntary male medical circumcision (VMMC) or male circumcision through traditional practices [22, 23]. Individuals who acquired HIV progressed through stages of acute, chronic, and late-stage HIV disease, with declining CD4+ T-cell counts throughout, unless effective treatment was initiated, which reconstituted CD4 count and reduced mortality and transmissibility [24]. Individuals on ART are assumed to be 96% less infectious. Treatment was allocated using an HIV care continuum beginning with HIV diagnosis and linkage to care, retention in care, and adherence to treatment [25]. Initial diagnosis and re-entry into care could occur as part of general HIV testing programs, antenatal HIV screening, screening of children born to HIV-positive mothers, or as part of care-seeking for symptoms in the late stage of disease [26]. Vertical transmission is modeled separately for peripartum and breastfeeding periods, with route-specific probabilities conditioned on maternal ART status (peripartum transmission risk of 15% without antiretrovirals vs 2% with; breastfeeding-associated risk of 13% per annum without antiretrovirals vs 1% with).

Additional model details are provided in Supplementary Appendix [27] and elsewhere [28].

### Model Calibration to Zambia

The model was calibrated to demographic and HIV epidemiological data from Zambia at the national and provincial levels using Parallel Simultaneous Perturbation Optimization [29], a hill-climbing optimization algorithm that parallelizes Spall's algorithm for computational efficiency. The 100 best-fitting parameter sets from calibration were selected for use in the analysis.

Calibrated parameter values and distributions, and comparison of model estimates to calibration target data, are provided in Supplementary Tables 5–7 and Supplementary Figures 1–9.

### Scenario Design

We aimed to develop a parsimonious yet representative model scenario for bilateral aid disruptions in Zambia, emphasizing the effects most likely to impact HIV transmission and mortality. Because the disruptions extended to electronic health systems and national data systems, it was not possible to directly measure changes in service provision. Therefore, informed by the Group Model Building method [30], co-authors engaged in iterative discussion toward a shared understanding of the main effects of the disruptions. Discussions took place among the co-authors of this study in February 2025 in Lusaka, Zambia.

Service impacts were based on Zambia Ministry of Health co-authors’ knowledge of HIV service financing in Zambia, implementing partners delivering services in each province, inter-dependencies between services, and available reserves of consumables and financial resources. Co-authors initially provided descriptive accounts of the effects of bilateral aid disruption. Through iterative discussion, the group enhanced the description with quantitative inputs and iterated toward estimates of the number or proportion of services no longer being rendered. Discussions were revisited in March 2025 to confirm the accuracy of initial estimates and revisit uncertainty ranges to be used in sensitivity analyses.

### Scenario Assumptions

Final assumptions regarding the impacts of funding disruptions focused on the categories of HIV care continuum and HIV prevention (Table 1). Effects on the HIV care continuum were assumed as follows: in 5 provinces employing USAID front-line workers (Luapula, Copperbelt, Northern, Muchinga, and Central Provinces), we assumed 40% of individuals who would be initiating or continuing on HIV treatment would lose access to treatment in January 2025. This represented the proportion of HIV care delivered in facilities predominantly staffed by USAID [31], as well as the proportion of HIV treatment commodities procured by PEPFAR [6]. A further 30% of care was provided in facilities co-staffed by USAID and Zambian government-employed healthcare workers; we assumed the latter cadre would absorb the duties of terminated workers. In sensitivity analysis, we included a pessimistic scenario of 70% ART access reduction in the 5 provinces, representing care in all facilities with USAID-employed providers, and an optimistic scenario of 10% reduction in ART access, representing greater back-filling of USAID roles by government-employed staff. This assumption is based on the estimated percentage of clinics affected by funding disruptions, rather than the percentage of clients. A limitation of this assumption is that it does not fully capture variation in client volume across facilities. However, because 5 provinces to which this assumption is applied are predominantly rural, clinic volumes are less variable than in settings with substantial urban populations. Therefore, uncertainty arising from variation in clinic volume is on par with other uncertainties regarding the extent of service disruption (represented in our sensitivity analysis).

**Table 1.** Model Assumptions on the Impact of Bilateral Aid Disruptions on HIV Treatment Programs and Prevention Services in Zambia

HIV Service Type	Baseline	Impact of Funding Disruption*	Range**	Reference
*HIV care continuum*				
Treatment access	No disruption in HIV care continuum occurs in 2025; HIV treatment coverage increases from 93.7% in 2025 to 95.0% by 2060 (see Supplementary Figure 10).	40% of new and returning clients in 5 provinces in Northern Zambia*** lose access to treatment.	10%–70% of new and returning clients in 5 of Zambia's 10 provinces*** lose access to treatment.	[31, 32]
Treatment attrition		20% per y.	10%–30% per y.	[33]
*HIV prevention services*				
Pre-exposure prophylaxis (PrEP)	Continue 2024 rate of 273 500 initiations per y, with average continuation of 3 m.	Only pregnant and breastfeeding women access PrEP, continuing at 2024 levels (46 516 initiations per y)	50%–80% reduction in relative to 2024, except for pregnant and breastfeeding women who continue 2024 levels (total of 97 000–180 000 initiations per y).	[6, 34]
Voluntary medical male circumcision (VMMC)	85% of adolescents aged 15–19 y.	100% reduction (no VMMC provision).	50%–80% reduction.	[35, 36]

^*^We assumed that the impact of PEPFAR funding pause would last for 3 m, 1 y, 4 y, or unabated.

^**^Range is used for 1-way sensitivity analysis.

^***^Luapula, Copperbelt, Northern, Muchinga, Central Provinces where UNAID provided front-line health workers.

In all 10 provinces, we assumed that rates of attrition from HIV treatment would increase from 5% per year to 20% per year. This was benchmarked based on the attrition rate observed prior to the implementation of appointment systems, defaulter tracing, multimonth dispensing of medications, and related interventions that were stopped following the aid disruption [33]. In sensitivity analysis, we included a pessimistic scenario of 30% attrition, representing the unique challenges of the abruptness of aid withdrawal creating circumstances not seen prior to implementation of health systems and care continuum strengthening, eg, loss of electronic health records in clinics that had implemented fully digital workflows and had no paper-based records on-site. We further included an optimistic scenario of 10% attrition, representing the potential for health systems resilience and continuation of some functions previously supported by PEPFAR.

We further considered the effects of aid disruptions on HIV prevention, particularly PrEP and VMMC services. Compared with 273 500 individuals who initiated oral PrEP in 2024, under the disruption only pregnant and breastfeeding women would receive PEPFAR-supported PrEP services, for an estimated 46 516 individuals accessing PrEP in 2025 [32]. All other PrEP was discontinued. All VMMC was discontinued [35]. However, men who were circumcised prior to the aid disruption continued to experience reduced rates of HIV acquisition, as the HIV prevention benefits of VMMC persist after the procedure [36]. Conservatively, we did not model the effects of reduced spending on other prevention services such as condom distribution, sexual health education, or behavior change promotion, as the effects of ceasing these services in the context of the aid disruption were not known. In sensitivity analysis, we explored optimistic scenarios of continuing to provide 20% and 50% the number of PrEP and VMMC services that were provided in 2025; we did not explore pessimistic assumptions because the services were stopped completely during the PEPFAR pause, with the exception of pregnant and breastfeeding women accessing PrEP.

We assumed all of the above-described effects would occur concurrently over the duration of the aid disruption, and varied the durations of the disruption: 3 months, 1 year, 4 years, or unabated. We simulate an unabated disruption not because we think it is likely that the disruption will persist for decades, but rather to demonstrate the upper bound of harm that could occur if the disruption is not remediated in a timely manner. At the end of a disruption, all HIV services were assumed to return to 2024 coverage levels and remain at those levels over the remainder of the analysis.

### Analysis

EMOD simulations were run on NYU Langone's Cray CS500 supercomputer. Each disruption scenario was run 100 times with different parameter values obtained from calibration (Supplementary Table 7), with simulations lasting through the year 2060. Outputs from simulations were analyzed using R version 4.2.1 with the library EMODAnalyzeR.

## RESULTS

Our modeled trajectories show HIV aid disruptions causing large increases in HIV-related deaths in Zambia. Between 2025 and 2060, a 3-month disruption was estimated to cause an additional 32 550 deaths, representing an 8.7% increase relative to no disruption, whereas a 4-year disruption would cause an additional 330 400 deaths over the same period (+89% compared with no disruption). Regardless of disruption duration, the largest percentage increase in deaths occurred in children, with an estimated 3922 additional child HIV deaths (+43%) from a 3-month disruption, compared with 29 320 additional child HIV deaths (+320%) from an unabated disruption. The largest numeric increases in deaths were among women, with 165 600 deaths (+88%) of women projected from a 4-year disruption (Table 2, Supplementary Table 8).

**Table 2.** Impact of Bilateral Aid Disruptions Lasting Different Durations (3 Months, 1 Year, 4 Years, or Unabated) on Additional HIV Deaths, Infections, and Person-years on Treatment in Zambia, 2025–2060

Disruption Duration	All	Men	Women	Children
Additional HIV Deaths				
3 M	32 550 (+8.7%)	12 695 (+7.3%)	15 782 (+8.4%)	3922 (+42.8%)
1 Y	115 080 (+30.9%)	45 897 (+26.3%)	57 339 (+30.5%)	11 778 (+128.5%)
4 Y	330 437 (+88.7%)	135 555 (+77.6%)	165 594 (+87.9%)	29 322 (+317.7%)
Unabated	1 591 774 (+427.2%)	618 122 (+353.9%)	790 933 (+419.4%)	183 387 (+1982.0%)
Additional HIV Infections				
3 M	54 863 (+13.1%)	20 278 (+12.7%)	27 807 (+11.7%)	6951 (+37.9%)
1 Y	195 414 (+46.6%)	71 140 (+44.1%)	99 167 (+41.6%)	25 411 (+137.9%)
4 Y	552 504 (+131.5%)	205 915 (+127.4%)	278 947 (+116.5%)	67 370 (+364.2%)
Unabated	3 296 117 (+783.5%)	1 365 738 (+844.2%)	1 545 515 (+643.5%)	382 736 (+2063.4%)
Addition Person-Y on HIV Treatment				
3 M	13 135 (0.0%)	−28 565 (−0.2%)	14 800 (+0.1%)	26 978 (+12.1%)
1 Y	−112 614 (−0.3%)	−150 327 (−1.2%)	−76 968 (−0.3%)	111 369 (+49.1%)
4 Y	−604 902 (−1.8%)	−499 580 (−3.9%)	−405 259 (−1.9%)	294 186 (+129.5%)
Unabated	−13 280 698 (−38.9%)	−4 945 958 (−38.6%)	−8 788 407 (−41.7%)	457 545 (+200.5%)

Disruptions begin in January 2025 and are compared to a scenario in which no disruption occurred. Values in parentheses indicate percentage change compared with no disruption. The results are stratified for men ages 15+, women ages 15+, and children ages 0–14.

**Figure 1.**
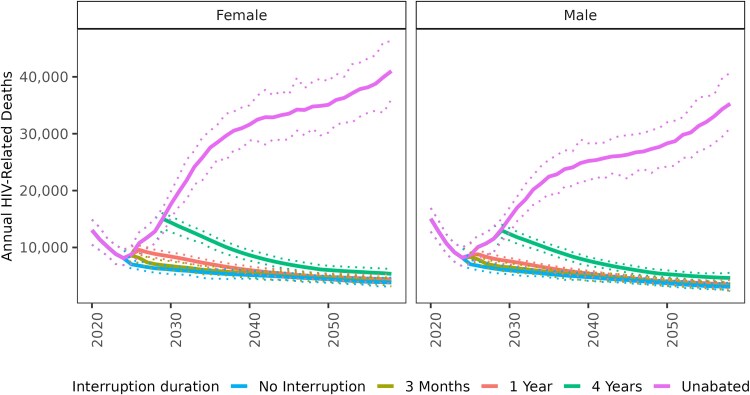
Impact of bilateral aid disruptions lasting different durations (3 m, 1 y, 4 y, or unabated) on annual HIV deaths in Zambia. Disruptions begin in January 2025. The results are reported for all ages and are stratified by sex.

If the disruption were limited to 3-month duration, mortality rates were projected to return to levels that would have been seen if there were no disruption (“baseline”) the following year. If the disruption lasted 1 year, mortality rates required 2 decades to return to baseline. If the disruption lasted for 4 years, mortality rates would not return to baseline for at least 4 decades (Figure 1).

**Figure 2.**
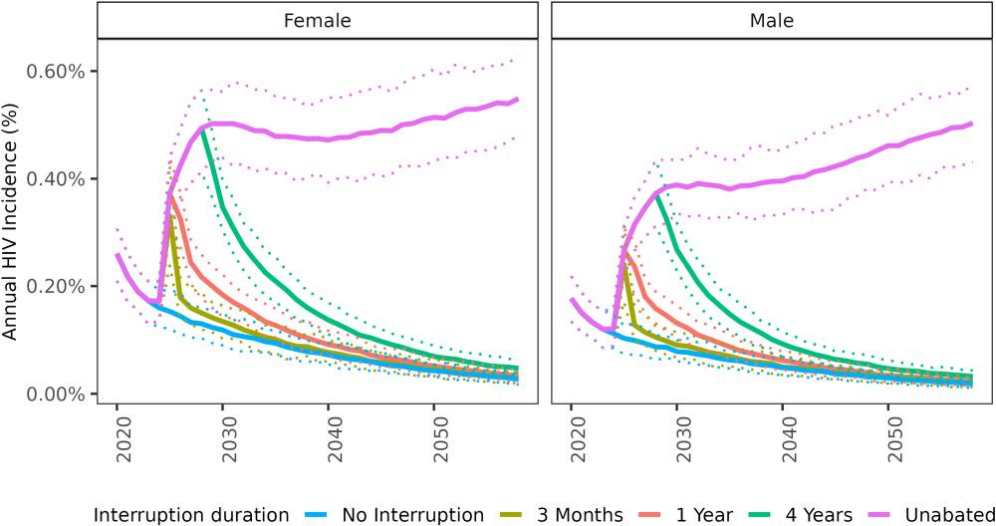
Impact of bilateral aid disruptions lasting different durations (3 m, 1 y, 4 y, or unabated) on annual HIV incidence rate (infections per 100 person-years) among adults ages 15 + in Zambia. Disruptions begin in January 2025. The results are stratified by sex.

HIV aid disruptions increased the number of new HIV infections acquired in Zambia. Effects were more immediate than those on mortality, with a 3-month aid disruption leading to 54 860 new infections (+13%), and a 4-year disruption leading to 552 500 new infections (+130%). A sharp spike in annual HIV incidence occurred during the disruption, with incidence dropping close to baseline levels after a 3-month or 1-year disruption, but remaining elevated well above baseline following a 4-year or unabated disruption (Figure 2).

Disruptions could either increase or decrease the number of person-years on HIV treatment, depending on the duration of the disruption (Table 2). While all disruptions led to lower HIV treatment coverage among PLHIV (Supplementary Figure 10), brief disruptions led to increases in new HIV infections exceeding increases in mortality, with overall more PLHIV requiring treatment once services resumed. For example, a 3-month disruption led to an additional 13 140 person-years on treatment over 2025–2060. Longer disruptions led to overall decreases in HIV treatment, but at the cost of extremely high mortality. Regardless of the duration of disruption, HIV treatment among children was projected to increase by 12% to 200%, reflecting a resurgence of HIV infections in the child population in which HIV had been declining.

**Figure 3.**
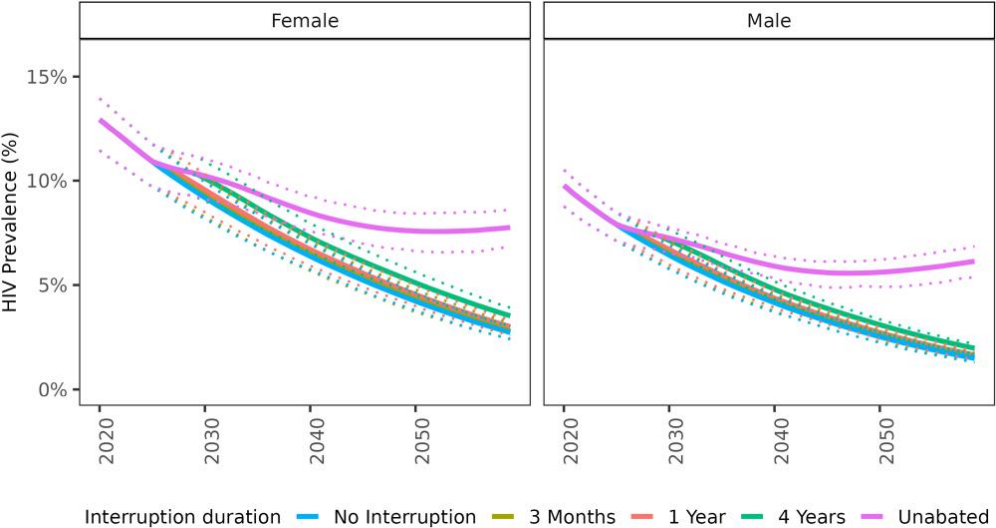
Impact of bilateral aid disruptions lasting different durations (3 m, 1 y, 4 y, or unabated) on HIV prevalence in Zambia. Disruptions begin in January 2025. The results are shown for adults ages 15 + and are stratified by sex.

Disruptions increased HIV prevalence, with the largest effects seen when disruptions continued for prolonged periods (Figure 3, Supplementary Table 9). A continuous disruption over 1 decade would lead to a roughly 1.25-fold increase in HIV prevalence among adults (men: 1.27x, women: 1.23x) and a 4.6-fold increase among children by 2035. A continuous disruption over 3 decades would lead to a roughly 2.75-fold increase in HIV prevalence among adults (men: 3.13x, women: 2.31x) and a 41.3-fold increase among children by 2055. By 2060, unabated disruption will lead to a 3.46-fold increase in HIV prevalence (4.20x in men, 2.89x in women, and 63.08x in children).

In sensitivity analysis (Supplementary Figure 11), the results were highly sensitive to uncertainties regarding clinic closures in Luapula, Copperbelt, Northern, Muchinga, and Central Provinces, where USAID employed front-line health providers. Under more pessimistic assumptions that 70% of HIV treatment would be disrupted in these provinces, a 3-month disruption added 59 300 HIV acquisitions (+14%) and 37 420 HIV deaths (+10%), while a 4-year disruption added 567 500 HIV acquisitions (+130%) and 348 200 HIV deaths (+94%). Under more optimistic assumptions that only 10% of HIV treatment would be disrupted in these provinces, a 3-month disruption added 48 350 HIV acquisitions (+12%) and 28 790 HIV deaths (+7.7%), while a 4-year disruption added 541 000 HIV acquisitions (+130%) and 313 500 HIV deaths (+84%). The results were also highly sensitive to the effects of nation-wide loss of health systems and care continuum strengthening services, such as electronic health records, appointment systems, and defaulter tracing. Under a pessimistic assumption that treatment attrition rates would increase to 30% per year (higher than the 20% per year observed prior to the introduction of these measures), a 3-month disruption added 77 280 HIV acquisitions (+18%) and 43 470 HIV deaths (+12%), while a 4-year disruption added 707 100 HIV acquisitions (+170%) and 424 300 HIV deaths (+110%). Under an optimistic assumption that treatment attrition rates would increase to 10% per year (lower than the 20% per year observed prior to the introduction of these measures), a 3-month disruption added 40 030 HIV acquisitions (+9.9%) and 22 940 HIV deaths (+6.2%), while an unabated disruption added 369 800 HIV acquisitions (+88%) and 217 500 HIV deaths (+58%). The results were comparatively less sensitive to uncertainties regarding HIV prevention services (Supplementary Figure 11).

## DISCUSSION

Our modeling shows that bilateral aid disruptions are causing a resurgence in HIV-related deaths and new HIV infections in Zambia. If disruptions continue unabated, Zambia could experience over 1 million deaths among adults, and nearly a quarter-million deaths among children by 2060 (a 20-fold increase compared with no disruption). New HIV infections would resurge, with over 3 million additional infections by 2060, and HIV prevalence would more than double, with the largest fold increases among children. HIV mortality could return to baseline if disruptions are limited to 3-month duration, but would take over 2 decades to return to baseline if disruptions last a year or more. However, even a 3-month disruption—which, as of this manuscript's submission, appears to be a best-case scenario—was estimated to cause over 30 000 additional deaths and over 50 000 additional HIV acquisitions in Zambia.

Our findings emphasize the importance of sustaining commitments on HIV epidemic control in low-resource global settings. As a transmissible disease, HIV is prone to resurging when services are disrupted, reversing hard-won gains in HIV epidemic control. Because the largest number of additional HIV infections is estimated to occur among women, mother-to-child HIV transmission will increase, extending the HIV crisis to future generations. Our findings are largely in line with descriptive forecasts of the HIV bilateral aid disruption published to date, and our short-term projections align well with a prior study estimating the impact of a 3- or 6-month disruption to HIV treatment during the SARS-CoV-2 pandemic [37]. At the time of writing, few quantitative estimates of the aid disruption's impacts have been published. One study in South Africa reported numerically large but proportionally smaller impacts of service disruptions [38], likely because a smaller proportion of South Africa's HIV program is PEPFAR-supported. Partnerships between health ministries and mathematical modelers, such as ours, are likely to continue to surface the profound impacts of PEPFAR disruptions in low- and middle-income countries.

Beyond the modeled scenarios, several real-world factors could amplify or attenuate the projected number of HIV-related infections and deaths. For example, the speed and effectiveness of governmental or donor responses—such as the reallocation of domestic funds or rapid engagement of other NGOs or donors to fill funding gaps—could help mitigate service disruptions. Behavioral responses may also influence outcomes. Some individuals could seek care through alternative pathways (eg, private pharmacies) and bear some of the cost of HIV services, although this approach risks widening health disparities by making care unaffordable to the most vulnerable. Lastly, shifts in sexual behavior patterns, whether toward greater or lesser HIV risk, could alter the underlying transmission dynamics over time. Our projections are based on the simplistic assumption of time-invariant disruption scenarios, assuming no major changes to service delivery or individual behavior while a disruption is in effect, and should be interpreted with caution given the presently dynamic state of global health.

To reduce the impact of aid withdrawal and resulting disruptions to HIV service delivery, the Zambia Ministry of Health, together with cooperating and development partners, explored adaptive strategies to shift the HIV response toward greater domestic ownership and sustainability. This included creating a national sustainability roadmap and updating HIV guidelines to a minimum package of core HIV services considered sustainable and cost-effective within current health financing constraints. Increasing private sector involvement through market strategies and expanding the role of the national health insurance scheme could help diversify HIV financing. Supply chain resilience could be further strengthened by restructuring the Zambia Medicines and Medical Supplies Agency, along with improved stock monitoring in collaboration with UN agencies. The Ministry is also working to fully integrate and harmonize HIV services into existing health systems at all levels, including transitioning healthcare workers previously supported by PEPFAR to the government payroll.

Our study has several important limitations. First, assumptions regarding the effects of funding disruptions on HIV service provision were simplistic in order to define scenarios with clarity and parsimony in the absence of direct health systems data. Because electronic health records and other data systems were inaccessible due to aid disruptions, we relied on health authorities’ knowledge of HIV service implementation and its reliance on specific international aid resources and worker cadres. We believe these to be reasonable approximations of ongoing service disruptions, but approximations nonetheless, as reflected in the wide uncertainty ranges that we explored. Estimates should be updated if and when granular local data become available. Second, our study did not include the effects of discontinuing some HIV services, such as behavior change messaging and condom distribution, as well as systems-level aid such as health systems strengthening and strategic information. The limited set of service disruptions that we included led to very large increases in mortality and HIV epidemic resurgence, which could be even larger if additional disruptions were incorporated. Third, we assumed that reductions in HIV services would continue at the same level until the end of the disruption. In reality, partial service restorations may occur if aid is partially restored, increased government spending is allocated toward HIV programs, or health systems adapt to lost resources. Conversely, service disruptions could become more severe over time as budgets and commodity stocks are depleted, mental health and well-being of healthcare workers is eroded, and other destabilizing effects of aid withdrawal. In Zambia and many other settings, HIV programs serve as key entry points for diagnosis and treatment of other conditions such as tuberculosis, indicating that disruption could have ripple effects beyond HIV. Moreover, diverting resources to HIV services may exacerbate existing funding gaps in areas such as maternal and child health, primary health care, or family planning [39]. The geographic distribution of disruptions could also change if there is re-allocation of funds, commodities, and—albeit to a lesser extent—healthcare personnel. Analysis of impacts should be revisited as these trends emerge. Finally, service disruptions likely have myriad indirect health effects not captured in this study. These may include eroding of service quality due to abrupt increases in healthcare workloads and scope-of-work, and rises in orphanhood, lost livelihoods, and other factors that were prevalent during the mid-2000's peak of the AIDS crisis.

## CONCLUSIONS

As a widely disseminated virus that is almost universally fatal if left untreated, HIV requires continuous public health effort to avoid resurgences. Prior to 2025, HIV incidence and mortality were steadily declining in Zambia through its predominantly PEPFAR-supported HIV program. We found that abrupt funding withdrawals leading to service disruptions, even if brief, could lead to HIV epidemic resurgence, while prolonged disruptions could reverse decades of gains and resurrect the AIDS crisis. Our findings underscore the need for gradual, planned, and monitored changes to bilateral aid that enable sustainable change without disrupting service provision, especially for those living with HIV. The Zambian HIV program now requires redoubled efforts to recover lost ground and resume progress toward epidemic control.

## Supplementary Material

ofaf511_Supplementary_Data

## Supplementary Data

Supplementary materials are available at *Open Forum Infectious Diseases* online. Consisting of data provided by the authors to benefit the reader, the posted materials are not copyedited and are the sole responsibility of the authors, so questions or comments should be addressed to the corresponding author.
