# Supplementary material for: Impacts of US Bilateral Aid Disruptions on HIV Resurgence in Zambia: A Mathematical Modeling Study
Source: Open Forum Infect Dis. 2025 Sep 11;12(9):ofaf511. doi: 10.1093/ofid/ofaf511 (PMC12461847; doi:10.1093/ofid/ofaf511)
Supplement: ofaf511_Supplementary_Data [file ofaf511_supplementary_data.docx]

**Online Supplementary Appendix**

*Accompanying the manuscript:*

*Impacts of United States bilateral aid disruptions on HIV resurgence in Zambia: a mathematical modeling study*

**Contents**

[Empiric Data for Model Calibration and Validation 3](#_Toc194659612)

[Supplementary Table 1: Population size by gender, province, and age group in 2010 in Zambia^¥^ 3](#_Toc194659613)

[Supplementary Table 2: Age-specific population fertility rate^¥^ 3](#_Toc194659615)

[Supplementary Table 3: Age-specific mortality rates in Zambia 1950-2050 among men^¥^ 4](#_Toc194659616)

[Supplementary Table 4: Age Specific mortality rates in Zambia 1950-2050 among women 5](#_Toc194659617)

[Supplementary Table 5: HIV prevalence data by age and sex from population-based surveys for model calibration in Zambia^¥^ 6](#_Toc194659618)

[Supplementary Table 6: Antiretroviral therapy coverage by age/sex from population-based surveys for model calibration, Zambia^¥^ 6](#_Toc194659619)

[Model Calibration and Fit 7](#_Toc194659620)

[Supplementary Table 7: Calibrated model parameters with initial values, allowable ranges, and final calibrated values. 7](#_Toc194659621)

[Fit to Prevalence 9](#_Toc194659622)

[Supplementary Figure 1: Model fit to prevalence for ages 15 to 50 9](#_Toc194659623)

[Supplementary Figure 2: Model fit to prevalence for ages 45 to 50 9](#_Toc194659624)

[Supplementary Figure 3: Model fit to prevalence for ages 40 to 45 10](#_Toc194659625)

[Supplementary Figure 4: Model fit to prevalence for ages 35 to 40 10](#_Toc194659626)

[Supplementary Figure 5: Model fit to prevalence for ages 30 to 35 11](#_Toc194659627)

[Supplementary Figure 6: Model fit to prevalence for ages 25 to 30 11](#_Toc194659628)

[Supplementary Figure 7: Model fit to prevalence for ages 20 to 25 12](#_Toc194659629)

[Supplementary Figure 8: Model fit to prevalence for ages 15 to 20 12](#_Toc194659630)

[Fit to ART Coverage 13](#_Toc194659631)

[Supplementary Figure 9: ART Coverage for ages 15 to 64 13](#_Toc194659632)

[Supplementary Results 14](#_Toc194659633)

[Supplementary Figure 10: Impact of bilateral aid disruptions lasting different durations (three months, one year, four years, or unabated) on HIV treatment coverage (percent of HIV-positive individuals receiving treatment) in Zambia. 14](#_Toc194659634)

[Supplementary Table 8: Impact of an unabated disruption of bilateral aid on HIV prevalence overall and among men, women, and children in Zambia. 15](#_Toc194659635)

[Supplementary Figure 11: Sensitivity of HIV-related deaths, HIV infections, and person-years on ART to key assumptions about unabated aid disruptions. 16](#_Toc194659637)

[Additional EMOD Specifications 17](#_Toc194659638)

# Empiric Data for Model Calibration and Validation

**Supplementary Table 1: Population size by gender, province, and age group in 2010 in Zambia¥**

|  | Age Group | Central | Copper-belt | Eastern | Luapula | Lusaka | Muchinga | Northern | North-western | Southern | Western |
| --- | --- | --- | --- | --- | --- | --- | --- | --- | --- | --- | --- |
| Women | 0 - 4 | 114,920 | 145,335 | 143,858 | 91,707 | 166,612 | 66,612 | 105,140 | 68,894 | 146,021 | 82,181 |
|  | 5 - 9 | 98,337 | 127,560 | 123,330 | 77,378 | 141,480 | 57,508 | 88,457 | 57,535 | 122,514 | 67,856 |
|  | 10 - 14 | 92,366 | 135,633 | 109,630 | 68,052 | 144,849 | 48,861 | 75,170 | 51,046 | 110,306 | 59,649 |
|  | 15 - 19 | 79,160 | 130,937 | 89,331 | 55,308 | 136,825 | 40,228 | 61,600 | 42,097 | 96,123 | 50,890 |
|  | 20 - 24 | 61,246 | 101,258 | 72,341 | 44,181 | 127,074 | 32,945 | 50,111 | 32,285 | 76,838 | 43,096 |
|  | 25 - 29 | 51,085 | 89,028 | 62,578 | 39,718 | 114,226 | 28,349 | 43,916 | 28,811 | 64,505 | 37,087 |
|  | 30 - 34 | 39,109 | 65,734 | 46,075 | 29,544 | 86,143 | 19,793 | 30,354 | 20,857 | 49,591 | 27,881 |
|  | 35 - 39 | 31,091 | 51,437 | 38,213 | 24,154 | 61,698 | 16,637 | 26,136 | 16,790 | 37,711 | 21,957 |
|  | 40 - 44 | 21,543 | 35,720 | 26,465 | 17,506 | 37,148 | 11,620 | 18,095 | 12,186 | 26,462 | 16,078 |
|  | 45 - 49 | 18,373 | 31,112 | 22,771 | 14,702 | 28,604 | 10,024 | 16,027 | 9,914 | 21,447 | 14,143 |
|  | 50 - 54 | 14,681 | 25,005 | 17,711 | 11,789 | 21,275 | 7,547 | 11,884 | 7,466 | 16,478 | 12,264 |
|  | 55 - 59 | 9,657 | 16,443 | 12,410 | 8,247 | 13,537 | 5,095 | 8,294 | 5,118 | 10,199 | 8,444 |
|  | 60 - 64 | 9,097 | 12,850 | 13,447 | 7,782 | 10,274 | 5,490 | 8,076 | 4,912 | 9,697 | 8,637 |
|  | 65+ | 17,981 | 22,378 | 29,821 | 13,270 | 18,482 | 11,076 | 15,713 | 10,992 | 22,375 | 19,306 |
| Men | 0 - 4 | 114,920 | 145,335 | 143,858 | 91,707 | 166,612 | 66,612 | 105,140 | 68,894 | 146,021 | 82,181 |
|  | 5 - 9 | 98,337 | 127,560 | 123,330 | 77,378 | 141,480 | 57,508 | 88,457 | 57,535 | 122,514 | 67,856 |
|  | 10 - 14 | 92,366 | 135,633 | 109,630 | 68,052 | 144,849 | 48,861 | 75,170 | 51,046 | 110,306 | 59,649 |
|  | 15 - 19 | 79,160 | 130,937 | 89,331 | 55,308 | 136,825 | 40,228 | 61,600 | 42,097 | 96,123 | 50,890 |
|  | 20 - 24 | 61,246 | 101,258 | 72,341 | 44,181 | 127,074 | 32,945 | 50,111 | 32,285 | 76,838 | 43,096 |
|  | 25 - 29 | 51,085 | 89,028 | 62,578 | 39,718 | 114,226 | 28,349 | 43,916 | 28,811 | 64,505 | 37,087 |
|  | 30 - 34 | 39,109 | 65,734 | 46,075 | 29,544 | 86,143 | 19,793 | 30,354 | 20,857 | 49,591 | 27,881 |
|  | 35 - 39 | 31,091 | 51,437 | 38,213 | 24,154 | 61,698 | 16,637 | 26,136 | 16,790 | 37,711 | 21,957 |
|  | 40 - 44 | 21,543 | 35,720 | 26,465 | 17,506 | 37,148 | 11,620 | 18,095 | 12,186 | 26,462 | 16,078 |
|  | 45 - 49 | 18,373 | 31,112 | 22,771 | 14,702 | 28,604 | 10,024 | 16,027 | 9,914 | 21,447 | 14,143 |
|  | 50 - 54 | 14,681 | 25,005 | 17,711 | 11,789 | 21,275 | 7,547 | 11,884 | 7,466 | 16,478 | 12,264 |
|  | 55 - 59 | 9,657 | 16,443 | 12,410 | 8,247 | 13,537 | 5,095 | 8,294 | 5,118 | 10,199 | 8,444 |
|  | 60 - 64 | 9,097 | 12,850 | 13,447 | 7,782 | 10,274 | 5,490 | 8,076 | 4,912 | 9,697 | 8,637 |
|  | 65+ | 17,981 | 22,378 | 29,821 | 13,270 | 18,482 | 11,076 | 15,713 | 10,992 | 22,375 | 19,306 |

^¥^Zambia 2010 Census of Population and Housing

**Supplementary Table 2: Age-specific population fertility rate**^¥^

| **Years** | **15-19** | **20-24** | **25-29** | **30-34** | **35-39** | **40-44** | **45-49** |
| --- | --- | --- | --- | --- | --- | --- | --- |
| 1950-1955 | 171.9 | 282.0 | 261.3 | 240.7 | 211.5 | 140.4 | 42.1 |
| 1955-1960 | 175.8 | 288.3 | 267.2 | 246.1 | 216.2 | 143.5 | 43.0 |
| 1960-1965 | 182.1 | 298.7 | 276.8 | 255.0 | 224.0 | 148.7 | 44.6 |
| 1965-1970 | 188.5 | 309.1 | 286.5 | 263.9 | 231.9 | 153.9 | 46.2 |
| 1970-1975 | 189.1 | 310.2 | 287.5 | 264.8 | 232.6 | 154.5 | 46.3 |
| 1975-1980 | 187.9 | 308.1 | 285.5 | 263.0 | 231.1 | 153.4 | 46.0 |
| 1980-1985 | 178.3 | 292.4 | 271.0 | 249.6 | 219.3 | 145.6 | 43.7 |
| 1985-1990 | 171.0 | 312.4 | 295.9 | 253.2 | 190.7 | 83.3 | 28.5 |
| 1990-1995 | 162.7 | 297.2 | 281.5 | 240.9 | 181.4 | 79.2 | 27.1 |
| 1995-2000 | 143.7 | 282.3 | 274.1 | 236.4 | 178.3 | 84.6 | 30.6 |
| 2000-2005 | 143.3 | 268.9 | 258.1 | 235.5 | 187.4 | 88.3 | 28.5 |
| 2005-2010 | 122.0 | 273.3 | 267.3 | 229.0 | 176.9 | 86.8 | 24.6 |
| 2010-2015 | 103.3 | 267.3 | 265.0 | 210.3 | 152.4 | 71.8 | 19.9 |
| 2015-2020 | 77.5 | 272.4 | 278.7 | 195.2 | 129.2 | 60.7 | 14.6 |
| 2020-2025 | 65.5 | 264.8 | 275.4 | 182.9 | 115.9 | 54.3 | 12.1 |
| 2025-2030 | 55.5 | 256.4 | 271.0 | 171.8 | 104.4 | 48.6 | 10.1 |
| 2030-2035 | 47.3 | 247.2 | 265.7 | 161.7 | 94.5 | 43.6 | 8.5 |
| 2035-2040 | 40.5 | 237.9 | 260.0 | 152.8 | 86.0 | 39.3 | 7.1 |
| 2040-2045 | 35.0 | 228.5 | 254.1 | 145.0 | 78.7 | 35.5 | 6.0 |
| 2045-2050 | 30.4 | 219.3 | 248.1 | 138.4 | 72.6 | 32.3 | 5.1 |
| 2050-2055 | 26.6 | 210.0 | 242.0 | 132.6 | 67.4 | 29.5 | 4.4 |
| 2055-2060 | 23.5 | 201.1 | 236.1 | 127.9 | 63.0 | 27.1 | 3.8 |
| 2060-2065 | 20.8 | 192.1 | 230.0 | 123.8 | 59.3 | 25.0 | 3.3 |
| 2065-2070 | 18.7 | 183.7 | 224.3 | 120.6 | 56.3 | 23.2 | 2.9 |
| 2070-2075 | 16.8 | 175.1 | 218.4 | 117.8 | 53.7 | 21.5 | 2.5 |
| 2075-2080 | 15.3 | 167.1 | 212.9 | 115.9 | 51.7 | 20.2 | 2.2 |
| 2080-2085 | 14.0 | 159.0 | 207.2 | 114.4 | 50.0 | 19.0 | 2.0 |
| 2085-2090 | 13.0 | 151.2 | 201.9 | 113.4 | 48.7 | 17.9 | 1.7 |
| 2090-2095 | 12.0 | 143.5 | 196.5 | 112.9 | 47.8 | 17.0 | 1.6 |
| 2095-2100 | 11.3 | 135.9 | 191.1 | 112.8 | 47.1 | 16.1 | 1.4 |

^¥^2015 United Nations World Population Prospects

**Supplementary Table 3: Age-specific mortality rates in Zambia 1950-2050 among men^¥^**

|  | Age Specific Mortality Rates | | | | | | |
| --- | --- | --- | --- | --- | --- | --- | --- |
| **Year** | 15-19 | 20-24 | 25-29 | 30-34 | 35-39 | 40-44 | 45-49 |
| **1950-1955** | 0.006199 | 0.008782 | 0.009304 | 0.009939 | 0.011253 | 0.013604 | 0.016357 |
| **1955-1960** | 0.005776 | 0.008187 | 0.008668 | 0.009258 | 0.010461 | 0.012652 | 0.015225 |
| **1960-1965** | 0.005386 | 0.007640 | 0.008083 | 0.008632 | 0.009734 | 0.011767 | 0.014146 |
| **1965-1970** | 0.005081 | 0.007226 | 0.007629 | 0.008146 | 0.009181 | 0.011088 | 0.013366 |
| **1970-1975** | 0.004682 | 0.006678 | 0.007033 | 0.007513 | 0.008463 | 0.010226 | 0.012392 |
| **1975-1980** | 0.004441 | 0.006384 | 0.006816 | 0.007320 | 0.008240 | 0.009804 | 0.011838 |
| **1980-1985** | 0.004508 | 0.006915 | 0.008538 | 0.009786 | 0.011312 | 0.011580 | 0.012806 |
| **1985-1990** | 0.004604 | 0.008014 | 0.013236 | 0.017624 | 0.021931 | 0.018999 | 0.017195 |
| **1990-1995** | 0.004516 | 0.008083 | 0.016345 | 0.024194 | 0.033568 | 0.028723 | 0.023526 |
| **1995-2000** | 0.004603 | 0.007224 | 0.015075 | 0.024252 | 0.036462 | 0.033729 | 0.027692 |
| **2000-2005** | 0.004800 | 0.006088 | 0.012258 | 0.020123 | 0.031510 | 0.030692 | 0.026542 |
| **2005-2010** | 0.003964 | 0.005122 | 0.007909 | 0.011383 | 0.016695 | 0.016480 | 0.015154 |
| **2010-2015** | 0.003598 | 0.004457 | 0.005899 | 0.008020 | 0.011331 | 0.011715 | 0.011573 |
| **2015-2020** | 0.003162 | 0.004151 | 0.005158 | 0.006668 | 0.009094 | 0.009797 | 0.009975 |
| **2020-2025** | 0.002896 | 0.004032 | 0.005157 | 0.006552 | 0.008389 | 0.008688 | 0.008846 |
| **2025-2030** | 0.002539 | 0.003727 | 0.004944 | 0.006344 | 0.008085 | 0.008004 | 0.007988 |
| **2030-2035** | 0.002117 | 0.003380 | 0.004496 | 0.005867 | 0.007525 | 0.007477 | 0.007341 |
| **2035-2040** | 0.001731 | 0.002975 | 0.003995 | 0.005215 | 0.006720 | 0.006818 | 0.006787 |
| **2040-2045** | 0.001557 | 0.002570 | 0.003481 | 0.004558 | 0.005844 | 0.006097 | 0.006241 |
| **2045-2050** | 0.001438 | 0.002340 | 0.003011 | 0.003940 | 0.005033 | 0.005391 | 0.005699 |

^¥^2015 United Nations World Population Prospects, minus estimated HIV related mortality

**Supplementary Table 4: Age Specific mortality rates in Zambia 1950-2050 among women**

|  | Age Specific Mortality Rates | | | | | | |
| --- | --- | --- | --- | --- | --- | --- | --- |
| Year | 15-19 | 20-24 | 25-29 | 30-34 | 35-39 | 40-44 | 45-49 |
| **1950-1955** | 0.005881 | 0.006813 | 0.007968 | 0.009224 | 0.010555 | 0.011723 | 0.012806 |
| **1955-1960** | 0.005410 | 0.006294 | 0.007361 | 0.008500 | 0.009718 | 0.010868 | 0.011914 |
| **1960-1965** | 0.005008 | 0.005854 | 0.006843 | 0.007882 | 0.009006 | 0.010139 | 0.011154 |
| **1965-1970** | 0.004668 | 0.005503 | 0.006405 | 0.007368 | 0.008417 | 0.009536 | 0.010521 |
| **1970-1975** | 0.004190 | 0.004990 | 0.005807 | 0.006662 | 0.007621 | 0.008749 | 0.009732 |
| **1975-1980** | 0.003899 | 0.004702 | 0.005492 | 0.006260 | 0.007144 | 0.008253 | 0.009237 |
| **1980-1985** | 0.003975 | 0.005305 | 0.006855 | 0.007402 | 0.007958 | 0.008541 | 0.009523 |
| **1985-1990** | 0.004170 | 0.007020 | 0.013088 | 0.014338 | 0.013807 | 0.011098 | 0.011405 |
| **1990-1995** | 0.004116 | 0.007732 | 0.019872 | 0.025314 | 0.025614 | 0.016908 | 0.014127 |
| **1995-2000** | 0.004155 | 0.007134 | 0.020667 | 0.030644 | 0.035577 | 0.023739 | 0.016800 |
| **2000-2005** | 0.004227 | 0.006027 | 0.017851 | 0.027699 | 0.035954 | 0.026396 | 0.018066 |
| **2005-2010** | 0.003294 | 0.004710 | 0.010333 | 0.014540 | 0.019129 | 0.015625 | 0.011939 |
| **2010-2015** | 0.002681 | 0.003591 | 0.006231 | 0.008397 | 0.010838 | 0.009859 | 0.008510 |
| **2015-2020** | 0.002298 | 0.002569 | 0.003789 | 0.005117 | 0.006650 | 0.007088 | 0.006667 |
| **2020-2025** | 0.002466 | 0.002206 | 0.002999 | 0.004559 | 0.006513 | 0.007326 | 0.006939 |
| **2025-2030** | 0.001974 | 0.002026 | 0.002197 | 0.002774 | 0.004217 | 0.005577 | 0.005878 |
| **2030-2035** | 0.001417 | 0.001760 | 0.001942 | 0.002030 | 0.002632 | 0.003965 | 0.004734 |
| **2035-2040** | 0.000996 | 0.001454 | 0.001702 | 0.001779 | 0.002064 | 0.003017 | 0.003869 |
| **2040-2045** | 0.000840 | 0.001173 | 0.001454 | 0.001587 | 0.001829 | 0.002615 | 0.003340 |
| **2045-2050** | 0.000744 | 0.001040 | 0.001241 | 0.001403 | 0.001658 | 0.002381 | 0.003049 |

^¥^2015 United Nations World Population Prospects, minus estimated HIV related mortality

**Supplementary Table 5: HIV prevalence data by age and sex from population-based surveys for model calibration in Zambia^¥^**

^¥^Demographic and Health Surveys (2001-02, 2007, 2013-14). Zambia Population-Based HIV Impact Assessment (2016, 2021).

**Supplementary Table 6: Antiretroviral therapy coverage by age/sex from population-based surveys for model calibration, Zambia^¥^**

^¥^Zambia Population-Based HIV Impact Assessment (2016, 2021)

# Model Calibration and Fit

**Supplementary Table 7: Calibrated model parameters with initial values, allowable ranges, and final calibrated values.**

| **Parameter name** | **Province(s)** | **Initial value & range** | **Calibrated values: mean (range)** |
| --- | --- | --- | --- |
| **Year of infection point in rising condom use** | All | 2001 (2001 – 2005) | 2001 (2001 – 2001) |
| **Condom use probability in transitory relationships** | Central | 0.90 (0.50 – 1.00) | 0.87 (0.76 – 1.00) |
|  | Copperbelt | 0.10 (0.00 – 0.55) | 0.12 (0.00 – 0.27) |
|  | Eastern | 1.00 (0.21 – 1.00) | 0.97 (0.75 – 1.00) |
|  | Luapula, Muchinga, and Northern | 0.70 (0.31 – 0.90) | 0.67 (0.54 – 0.78) |
|  | Lusaka | 0.10 (0.00 – 0.66) | 0.05 (0.00 – 0.24) |
|  | Northwestern | 0.70 (0.46 – 0.80) | 0.70 (0.61 – 0.77) |
|  | Southern | 0.70 (0.49 – 0.80) | 0.63 (0.56 – 0.72) |
|  | Western | 0.10 (0.00 – 0.80) | 0.09 (0.00 – 0.21) |
| **Condom use probability in informal relationships** | Central | 0.00 (0.00 – 0.60) | 0.05 (0.00 – 0.20) |
|  | Copperbelt | 0.00 (0.00 – 0.42) | 0.05 (0.00 – 0.15) |
|  | Eastern | 1.00 (0.02 – 1.00) | 0.98 (0.84 – 1.00) |
|  | Luapula, Muchinga, and Northern | 0.90 (0.41 – 1.00) | 0.96 (0.79 – 1.00) |
|  | Lusaka | 0.20 (0.00 – 0.60) | 0.09 (0.00 – 0.28) |
|  | Northwestern | 0.40 (0.26 – 0.60) | 0.42 (0.34 – 0.50) |
|  | Southern | 0.00 (0.00 – 0.60) | 0.02 (0.00 – 0.12) |
|  | Western | 0.10 (0.00 – 0.31) | 0.05 (0.00 – 0.16) |
| **Epidemic importation year** | Central | 1977 (1970 – 1990) | 1977 (1973 – 1980) |
|  | Copperbelt | 1977 (1970 – 1990) | 1979 (1975 – 1983) |
|  | Eastern | 1977 (1970 – 1990) | 1983 (1976 – 1988) |
|  | Luapula, Muchinga, and Northern | 1977 (1970 – 1990) | 1976 (1970 – 1982) |
|  | Lusaka | 1977 (1970 – 1990) | 1974 (1970 – 1980) |
|  | Northwestern | 1977 (1970 – 1990) | 1979 (1973 – 1983) |
|  | Southern | 1977 (1970 – 1990) | 1981 (1975 – 1985) |
|  | Western | 1977 (1970 – 1990) | 1977 (1973 – 1981) |
| **Epidemic importation fraction of high-risk groups** | Central | 0.10 (0.01 – 0.50) | 0.06 (0.01 – 0.19) |
|  | Copperbelt | 0.10 (0.01 – 0.50) | 0.08 (0.01 – 0.18) |
|  | Eastern | 0.10 (0.01 – 0.50) | 0.17 (0.01 – 0.28) |
|  | Luapula, Muchinga, and Northern | 0.10 (0.01 – 0.50) | 0.10 (0.01 – 0.27) |
|  | Lusaka | 0.10 (0.01 – 0.50) | 0.21 (0.06 – 0.31) |
|  | Northwestern | 0.10 (0.01 – 0.50) | 0.09 (0.02 – 0.21) |
|  | Southern | 0.10 (0.01 – 0.50) | 0.18 (0.02 – 0.29) |
|  | Western | 0.10 (0.01 – 0.50) | 0.15 (0.02 – 0.33) |

## Fit to Prevalence


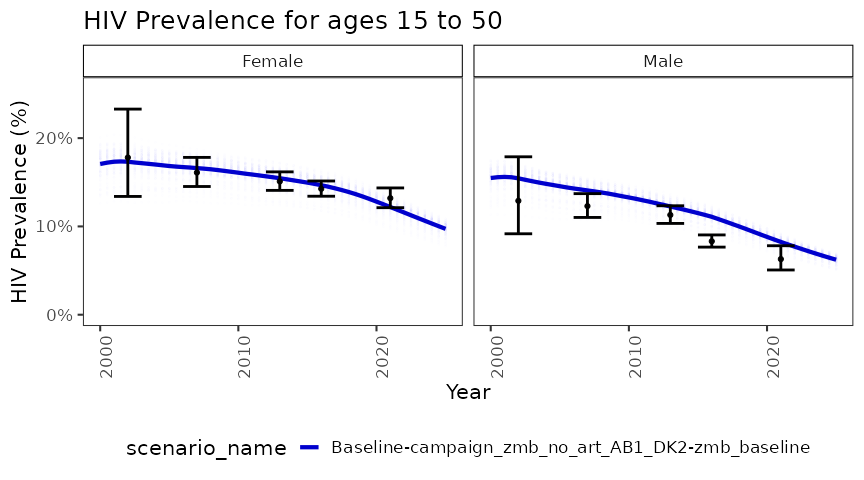


**Supplementary Figure 1: Model fit to prevalence for ages 15 to 50**


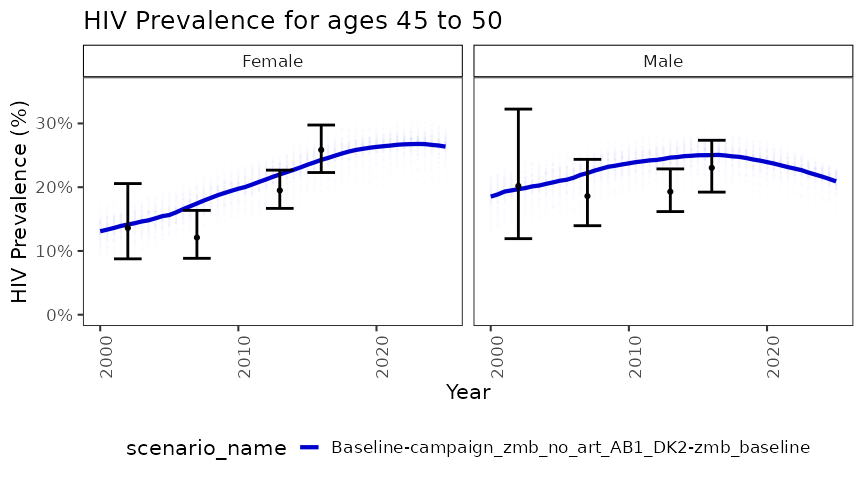


**Supplementary Figure 2: Model fit to prevalence for ages 45 to 50**


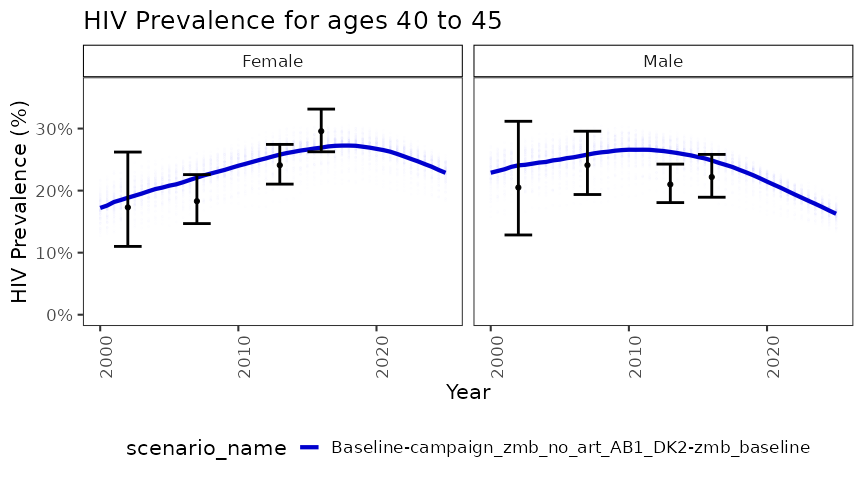


**Supplementary Figure 3: Model fit to prevalence for ages 40 to 45**


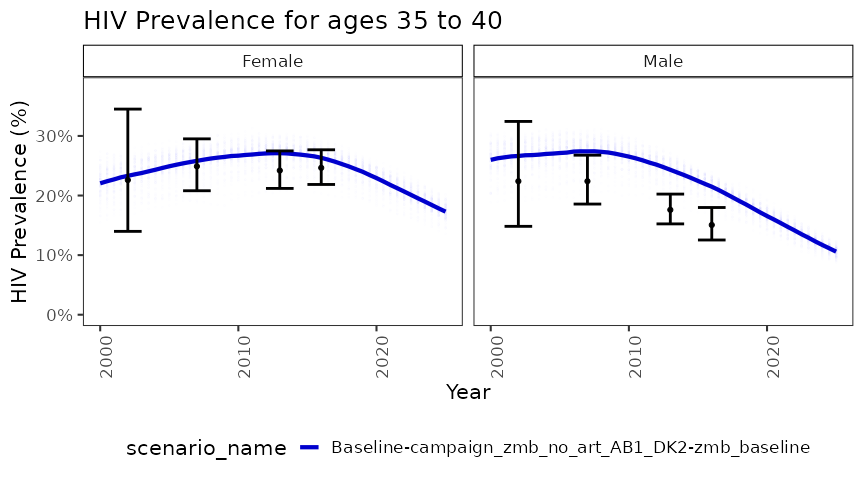


**Supplementary Figure 4: Model fit to prevalence for ages 35 to 40**


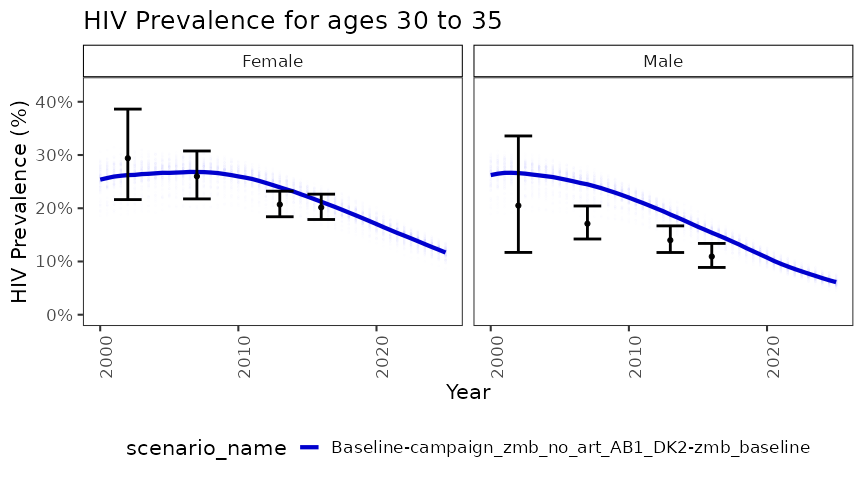


**Supplementary Figure 5: Model fit to prevalence for ages 30 to 35**


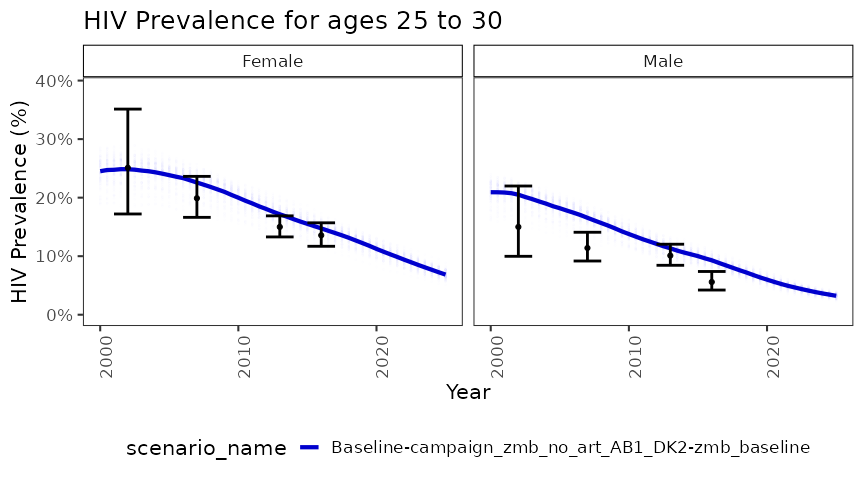


**Supplementary Figure 6: Model fit to prevalence for ages 25 to 30**


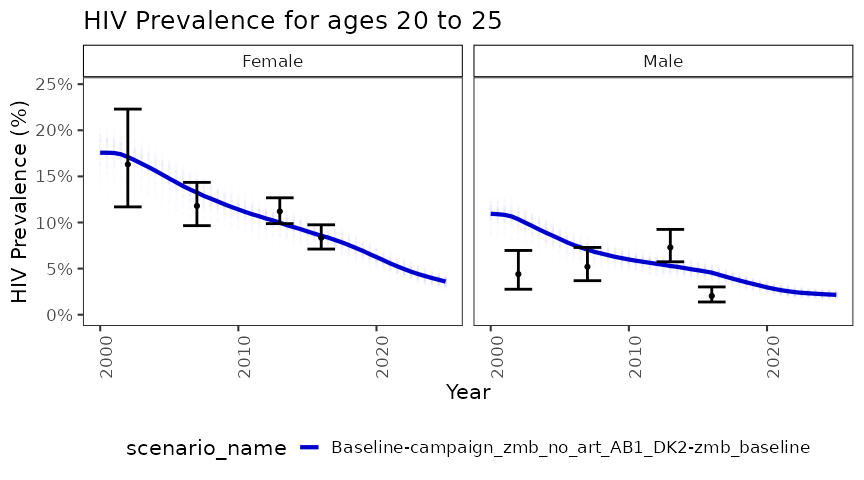


**Supplementary Figure 7: Model fit to prevalence for ages 20 to 25**


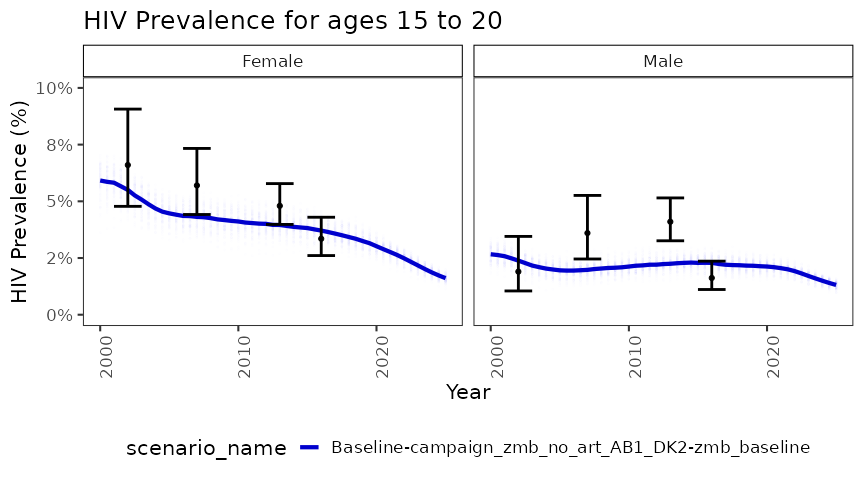


**Supplementary Figure 8: Model fit to prevalence for ages 15 to 20**

## Fit to ART Coverage


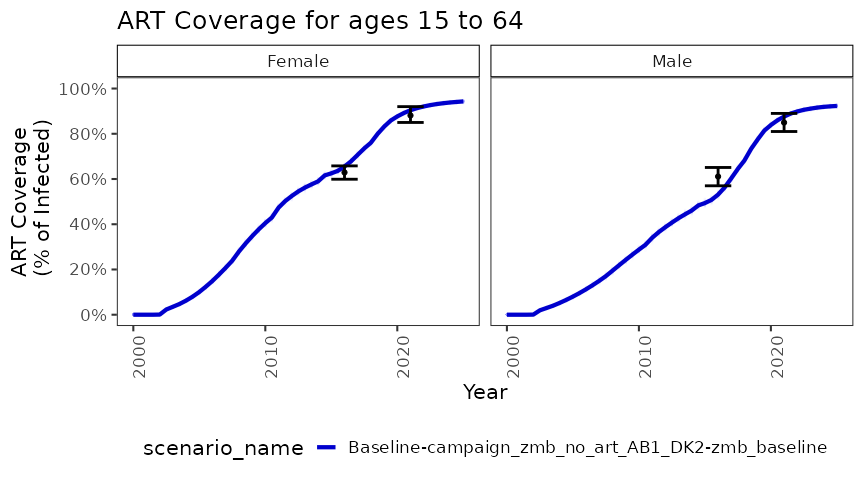


**Supplementary Figure 9: ART Coverage for ages 15 to 64**

# Supplementary Results


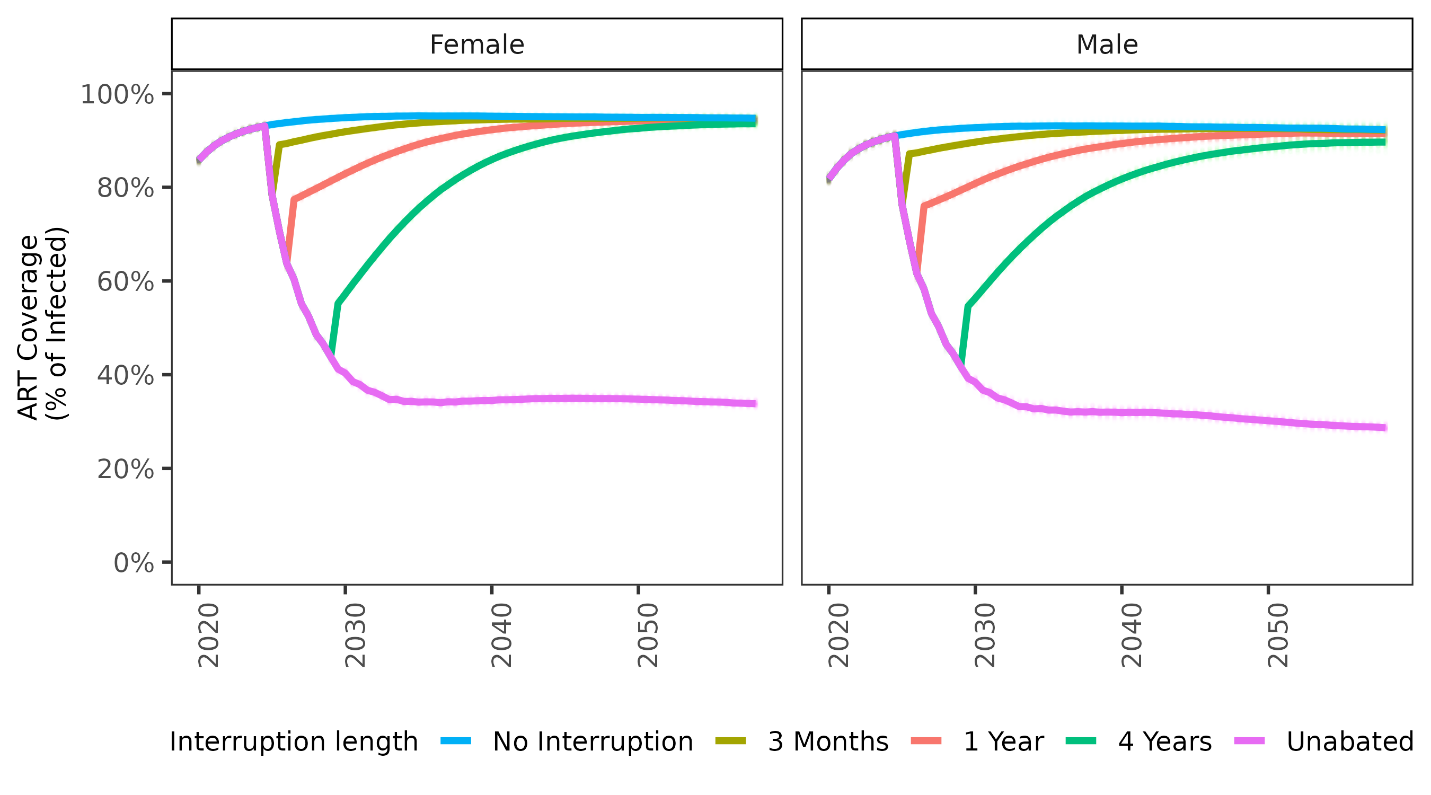


**Supplementary Figure 10:** **Impact of bilateral aid disruptions lasting different durations (three months, one year, four years, or unabated) on HIV treatment coverage (percent of HIV-positive individuals receiving treatment) in Zambia.** Disruptions begin in January 2025. Results are stratified by sex.

**Supplementary Table 8: Impact of an unabated disruption of bilateral aid on cumulative additional HIV deaths, infections, and person-years on treatment across time in Zambia.** HIV burden is reported cumulatively through the corresponding year, rather than over a constant time horizon as reported in the main manuscript. Values in parentheses indicate percentage change compared to no disruption. Results are stratified for men ages 15+, women ages 15+, and children ages 0–14.

|  | **Year** | **All** | **Men** | **Women** | **Children** |
| --- | --- | --- | --- | --- | --- |
| **Additional HIV Deaths** | **2025** | 5,011 (+21%) | 633 (+6%) | 740 (+8%) | 3,469 (+266%) |
|  | **2026** | 12,679 (+33%) | 2,729 (+15%) | 3,897 (+23%) | 5,799 (+277%) |
|  | **2030** | 77,877 (+83%) | 25,394 (+57%) | 33,546 (+77%) | 18,207 (+395%) |
|  | **2035** | 252,860 (+163%) | 92,513 (+126%) | 120,410 (+164%) | 38,186 (+595%) |
|  | **2055** | 1,341,278 (+386%) | 514,702 (+315%) | 662,872 (+385%) | 155,032 (+1711%) |
| **Additional HIV Infections** | **2025** | 27,577 (+113%) | 10,035 (+113%) | 13,106 (+103%) | 4,021 (+185%) |
|  | **2026** | 62,333 (+154%) | 23,078 (+156%) | 29,793 (+139%) | 8,656 (+241%) |
|  | **2030** | 264,728 (+261%) | 98,742 (+262%) | 125,174 (+229%) | 38,912 (+483%) |
|  | **2035** | 578,897 (+336%) | 219,720 (+339%) | 272,295 (+288%) | 83,716 (+714%) |
|  | **2055** | 2,709,921 (+690%) | 1,114,808 (+739%) | 1,257,405 (+569%) | 321,273 (+1781%) |
| **Addition Person-Years on HIV Treatment** | **2025** | -603,966 (-26%) | -243,099 (-27%) | -355,052 (-26%) | -9,873 (-22%) |
|  | **2026** | -1,066,635 (-31%) | -430,125 (-32%) | -629,305 (-31%) | -14,893 (-23%) |
|  | **2030** | -3,382,348 (-43%) | -1,359,164 (-43%) | -2,032,308 (-43%) | -18,916 (-15%) |
|  | **2035** | -6,430,149 (-48%) | -2,559,652 (-49%) | -3,931,660 (-49%) | 14,854 (+9%) |
|  | **2055** | -13,405,072 (-42%) | -5,068,201 (-42%) | -8,820,913 (-44%) | 374,989 (+167%) |

**Supplementary Table 9: Impact of an unabated disruption of bilateral aid on HIV prevalence overall and among men, women, and children in Zambia.** HIV prevalence is reported in different years. Prevalence to the left of the arrow is estimated in the absence of a disruption, while prevalence to the right of the arrow is estimated in the presence of disruption that continues through the end of the period of analysis. Values in parentheses indicate the fold increase in HIV prevalence due to the disruption.

| **Year** | **All** | **Men** | **Women** | **Children** |
| --- | --- | --- | --- | --- |
| **2025** | 5.4% → 5.5% (1.01x) | 7.8% → 7.9% (1.00x) | 10.8% → 10.9% (1.01x) | 0.4% → 0.4% (1.00x) |
| **2026** | 5.2% → 5.4% (1.02x) | 7.5% → 7.7% (1.02x) | 10.5% → 10.7% (1.02x) | 0.3% → 0.4% (1.03x) |
| **2030** | 4.6% → 5.2% (1.14x) | 6.4% → 7.2% (1.13x) | 9.1% → 10.2% (1.12x) | 0.2% → 0.3% (1.82x) |
| **2035** | 3.8% → 4.8% (1.27x) | 5.1% → 6.5% (1.27x) | 7.6% → 9.3% (1.23x) | 0.1% → 0.4% (4.57x) |
| **2055** | 1.7% → 4.5% (2.68x) | 1.9% → 5.9% (3.13x) | 3.3% → 7.6% (2.31x) | 0.0% → 0.5% (41.31x) |


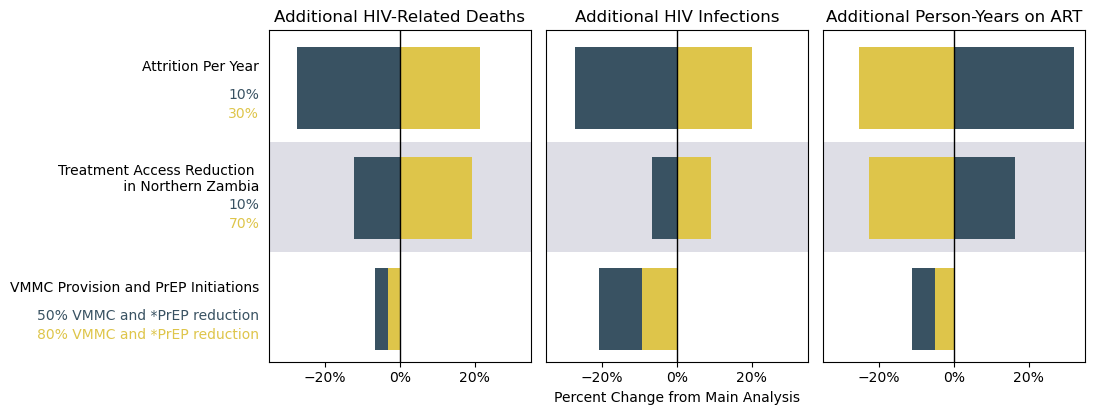


**Supplementary Figure 11: Sensitivity of HIV-related deaths, HIV infections, and person-years on ART to key assumptions about unabated aid disruptions.** *PrEP reduction does not include pregnant and breastfeeding women, who are assumed to experience no disruption to PrEP access.

# Additional EMOD Specifications

Additional specifications for the EMOD model can be found at https://docs.idmod.org/projects/emodpy-hiv/en/latest/overview.html

Topics covered in these documents include – but are not limited to – the pair formation algorithm, cascade of care modules, and HIV biology.
